# Midgut proteome of an argasid tick, *Ornithodoros erraticus*: a comparison between unfed and engorged females

**DOI:** 10.1186/s13071-015-1148-z

**Published:** 2015-10-12

**Authors:** Ana Oleaga, Prosper Obolo-Mvoulouga, Raúl Manzano-Román, Ricardo Pérez-Sánchez

**Affiliations:** Parasitology Laboratory, Instituto de Recursos Naturales y Agrobiología de Salamanca (IRNASA, CSIC), Cordel de Merinas, 40-52, 37008 Salamanca, Spain

**Keywords:** *Ornitodoros erraticus*, Soft tick, Midgut, Proteome, Blood digestion

## Abstract

**Background:**

The argasid tick *Ornithodoros erraticus* is the vector of African swine fever virus and of several *Borrelia* species that cause human relapsing fever in the Iberian Peninsula. The tick midgut is part of the ectoparasite-host interface and expresses proteins that are vital for the survival of the tick. Midgut proteins are therefore potential targets for drug and/or vaccine design aimed at the development of new strategies for tick control. Thus, the aim of this work was the characterization of the proteome of the *O. erraticus* midgut before and after a blood meal trying to elucidate the induced changes upon blood feeding.

**Methods:**

Midgut tissues from unfed and engorged *O. erraticus* females were dissected and proteins were fractionated by centrifugation and SDS-PAGE, and the corresponding gel pieces analysed by LC–MS/MS. The identified proteins were classified according to their Protein Class and Molecular Function and the differences between fed and unfed specimens were analysed.

**Results:**

Overall 555 tick proteins were identified: 414 in the midgut of the unfed specimens and 376 in the fed specimens, of which 235 were present in both groups. The proteins with catalytic, binding and structural functions were the most numerous and abundant, consistent with their role in the intracellular processing of the blood meal. The analysis of some groups of proteins putatively involved directly in blood meal digestion, including protein digestion (peptidase activity), iron metabolism, enzymes involved in oxidative stress and detoxification and membrane traffic and transport proteins, detected some differences between the fed and unfed ticks

**Conclusions:**

This work reports for the first time the collection and analysis of the midgut proteome of an argasid tick species and provides molecular information about the argasid machinery involved in blood digestion. This information represents a starting point for the identification and selection of new targets for the development of alternative control strategies.

**Electronic supplementary material:**

The online version of this article (doi:10.1186/s13071-015-1148-z) contains supplementary material, which is available to authorized users.

## Background

Ticks are blood-sucking arthropods that belong to two large families, Ixodidae (hard ticks) and Argasidae (soft ticks). They are of huge medical and veterinary importance not only because of the direct harm they cause to the host but also because they are the vectors of a large number of pathogens that affect livestock, pets, and humans [1, 2]. Among the argasid ticks, several species of the genus *Ornithodoros* are of special importance because they transmit pathogens that cause severe diseases such as human Tick-borne relapsing fever and African swine fever. Specifically, *Ornithodoros erraticus* is the main vector of these diseases in the Iberian Peninsula [3, 4].

The presence of this argasid in domestic and peridomestic environments contributes to the persistence of these diseases in endemic areas and also poses a constant threat for reintroduction, spread, and long term maintenance in areas from where they have been eradicated or where they have never existed. Thus, the prevention and control of these diseases would require the elimination of this argasid from synanthropic environments [5]. The application of chemical acaricides for the control of *O. erraticus* has severe drawbacks (acaricide resistance and contamination of the environment and animal products) and has proved to be inefficient [6–9]. These problems have stimulated the development of alternative methods for the control of this argasid tick, among which vaccines have emerged as the most promising, in particular those based on the concealed antigens of the tick midgut [1, 5]. In this sense, previous work carried out by our team reported immunization trials using a midgut surface exposed antigen in *O. erraticus* capable of significantly blocking feeding and reproduction performance in females and inducing lethal damages in the gut of nymphs fed on vaccinated animals [10, 11]. It was noted that such damages were mediated by host complement factors ingested with blood, in a similar way to that observed in the efficient hard tick vaccines based on the midgut Bm86 antigen [12, 13]. The *O. erraticus* antigen responsible for the observed protection remains to be identified, but these findings indicate that the midgut of argasid ticks could be an important source of candidate antigens for vaccines, in agreement with what has been proposed for ixodids by other authors [9, 14].

The tick midgut is the organ responsible for digesting the host’s blood and for absorbing the nutrients necessary for its survival and reproduction. Additionally, the tick midgut epithelium is a major physical barrier between the tick and the host defense mechanisms and also the initial site for pathogen infection being thus an important target for pathogen transmission blockage [15]. Accordingly, the midgut constitutes an important part of the host-tick-pathogen interface expressing proteins involved in vital functions for the tick and for tick invasion by pathogens ingested with the blood.

Unlike blood-feeding insects, which feed and digest blood rapidly in the neutral pH of the gut lumen, tick feeding is a slower process, and digestion takes place in the acidic intracellular compartment of the gut epithelium [16]. Moreover, the physiology of feeding and blood digestion differs substantially between hard and soft ticks [17–19]. In most argasid species, nymphs and adults take their blood meal rapidly, within minutes-hours, and then drop off the host. By contrast, ixodid ticks remain attached to their vertebrate host for long periods and feed continuously for days or even weeks [17].

In ixodid ticks, the digestive system, the blood digestion process and the digestion-associated histological modifications of the midgut epithelium have been addressed in many studies. Such studies have provided a solid understanding of how this tick family handles blood meals [16–20]. More recently, our understanding of these processes at molecular level has been substantially improved owing to the analysis of the midgut transcriptomes and proteomes of several ixodid species [21–26].

By contrast, the physiology and biochemistry of blood digestion in argasid ticks have been little studied and the information available is essentially limited to *O. moubata* [27–29]. To date, no argasid midgut proteome or transcriptome has been published.

In light of the foregoing, investigation of the *O. erraticus* midgut proteome might provide an in-depth understanding of the key cellular processes of the digestive physiology of argasids, affording valuable information about potential targets for drug and/or vaccine design aimed at the development of new strategies for tick control [23].

Thus, the aim of this work was the characterization of the proteome of the *O. erraticus* midgut before and after a blood meal trying to elucidate the induced changes upon blood feeding. To achieve this goal, midgut tissues from unfed and engorged *O. erraticus* females were dissected and proteins were fractionated by centrifugation and SDS-PAGE, and the corresponding gel pieces analysed by LC–MS/MS. Altogether, in fed and unfed ticks we identified 555 tick proteins, which were classified according to their Protein Class and Molecular Function. The differences between fed and unfed specimens are discussed.

## Methods

### Ticks and tick material

The colony of *O. erraticus* ticks is maintained in the laboratory of Animal Parasitology (IRNASA, CSIC) and was established from specimens captured in Salamanca province (western Spain). Ticks are fed regularly on rabbits and kept in a culture chamber at 28 °C, 85 % relative humidity and a 12 h light–dark cycle.

#### Ethical approval

Tick maintenance and all animal manipulation were done according to the rules from the Ethical and Animal Welfare Committee of the institution where the experiments were conducted (IRNASA, CSIC), following the corresponding EU rules and regulations.

### Preparation of midgut protein extracts for proteomic analyses

Midgut extracts were prepared from unfed females (unfed group) and from engorged females at 48 h post-feeding (fed group). To accomplish this, the ticks were dissected in sterile phosphate buffered saline (PBS) pH 7.4 at 4 °C and the midguts were removed and rinsed several times in PBS to eliminate host blood [10]. Batches of 50 midguts were suspended in fresh PBS containing a cocktail of proteinase inhibitors (Roche Diagnostics), homogenized on ice using an Ultra-Turrax T10 disperser (IKA-Werke), and then sonicated 6 times for 30 s/each. Tissue homogenates were centrifuged for 20 min at 10,000 × g and 4 °C to remove cellular debris, and the 10,000 xg supernatants were recovered and centrifuged for 1 h at 100,000 × g and 4 °C. These new supernatants were recovered and named S-0 and S-1, corresponding to the soluble fractions of midgut proteins from the unfed and fed ticks respectively. The pellets were re-suspended in PBS containing protease inhibitors and centrifuged once again for 1 h at 100,000 × g and 4 °C. The resulting new pellets were recovered and named P-0 and P-1, corresponding to the insoluble fractions of midgut proteins from unfed and fed ticks respectively. The protein concentrations in all these fractions were measured using the BCA Protein Assay Reagent kit (Thermo-Fisher). Samples were stored at −20 °C.

Samples of 20 μg from each fraction (S-0, S-1, P-0 and P-1) were mixed with 4x Laemmli buffer [30], heated to 90 °C for 3 min, and centrifuged at 10,000 xg for 4 min. The protein samples were then resolved by SDS-PAGE in 5–20 % gradient polyacrylamide gels and the gels were stained with Sypro Ruby (Bio-Rad) for protein visualization and image analysis (ChemiDoc System and Image Lab software, Bio-Rad) or with Coomassie Blue (Coomassie Blue R-25 0.125 %, methanol 50 %, acetic acid 10 %) for LC-MS/MS analysis (see below).

**Fig. 1:**
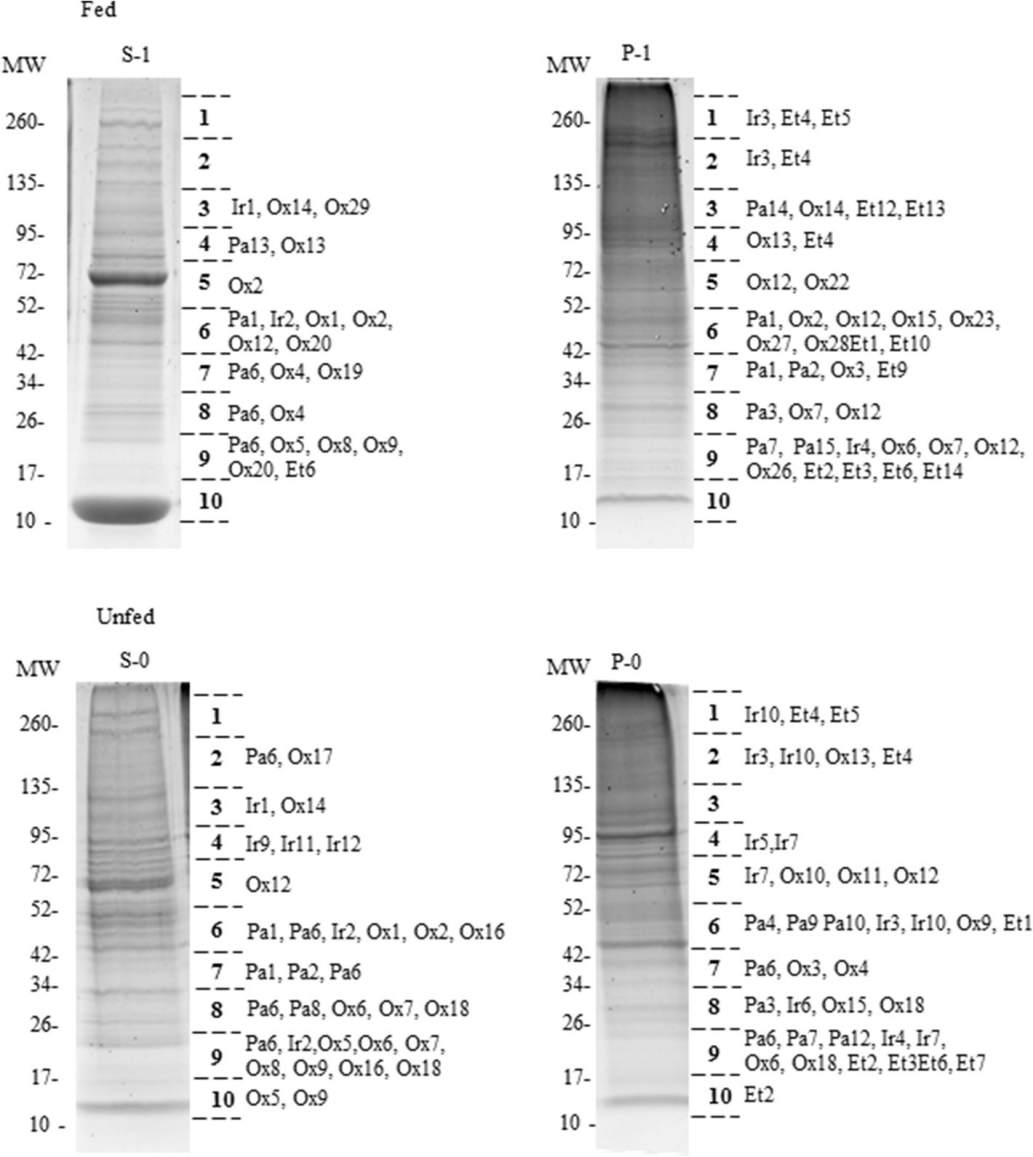
Sypro Ruby-stained 5-20 % polyacrylamide gel showing the protein fractions obtained from midgut homogenates of fed and unfed *Ornithodoros erraticus* ticks. Gel lanes were sliced into the 10 pieces indicated on the right, and the resulting gel fragments were digested with trypsin and analyzed by LC-MS/MS for protein identification. Some of the most interesting proteins identified in each gel fragment are indicated using custom alphanumeric codes. A description of these codes can be found in Table 2. S-1 and P-1, supernatant and pellet from midgut homogenates of fed ticks. S-0 and P-0, supernatant and pellet from midgut homogenates of unfed ticks

In the Coomassie blue-stained gels, each lane (corresponding to S-0, S-1, P-0 and P-1) was sliced into 10 pieces (see Fig. 1), which were sent to the SCSIE_University of Valencia Proteomics Unit, belonging to the ISCIII ProteoRed Proteomics Platform (Spain), for mass spectrometry analyses and protein identification.

### In-gel enzymatic digestion and liquid chromatography and tandem mass spectrometry (LC-MS/MS)

Each gel slice from the S-0, S-1, P-0 and P-1 fractions was subjected to enzymatic digestion and LC-MS/MS analysis. Briefly, the procedure was as follows: gel slices were conditioned with 50 % acetonitrile, dried, and digested with sequencing-grade trypsin (Promega) (20 ng/μl in 25 mM NH_4_HCO_3_) overnight at 37 °C. The reactions were stopped with 10 % trifluoroacetic acid at a final concentration of 0.1 %, and the supernatants were filtered through a 0.22 μm filter and dried by centrifugation in a vacuum. The concentration of peptides was estimated by UV spectrometry, assuming that a 1 mg/ml solution of proteins had an extinction coefficient of 1.1 absorbance units at 280 nm. A BSA plug was analysed in the same way to control the digestion process.

The peptides extracted after in-gel digestion were re-suspended in 5 μl of 5 % acetonitrile, 0.1 % trifluoroacetic acid, and 5 μl of the sample was loaded onto a trap column (NanoLC Column, 3 μ C18-CL, 350 μm × 0.5 mm, Eksigen) and desalted with 0.1 % trifluoroacetic acid at a flow rate of 3 μl/min for 5 min. The peptides were then loaded onto an analytical column (LC Column, 3 μ C18-CL, 75 μm × 25 cm, Eksigen) equilibrated in 5 % acetonitrile and 0.1 % formic acid. The peptides eluted were analysed with a nanoESI-Q-TOF mass spectrometer (5600 TripleTOF, ABSciex) in information-dependent acquisition mode, in which a 0.25-s TOF MS scan from 350 to 1250 m/z was performed, followed by 0.05-s product ion scans from 100 to 1500 m/z on the 50 most intense 2–5 charged ions.

### Database searching and protein identification

Searches were performed in the NCBInr_Metazoa (4,909,369 sequences) and NCBI EST_Acari (2,476,050 sequences) databases using the Mascot v2.2 (Matrix Science) search engine. Database searching was initially done individually for each gel piece and then jointly for each sample by combining the spectra from the 10 gel pieces into which the same sample had been sliced.

For the Mascot searches, the peak lists were generated directly from QSTAR wiff files by Mascot Daemon v. 2.2.2 (Matrix Science) with Sciex Analyst import filter options using the default parameters. Databases were searched using the following parameters: tryptic specificity, allowing one missed cleavage and a tolerance in the mass measurement of 70 ppm in MS mode and 0.6 Da for MS/MS ions. The carbamidomethylation of Cys was set as a fixed modification, and Met oxidation and Asn/Gln deamidation were set as variable modifications. The significance threshold was set at 0.05 and only proteins with at least two unique significant peptides were selected and shown in the results.

The relative abundance of a protein in the sample was quantified using the protein abundance index (PAI), which is defined as the number of observed peptides in the experiment divided by the number of observable tryptic peptides for each protein within a given mass range of the mass spectrometer employed [31]. The PAI was modified exponentially to give emPAI, the exponential form of PAI minus one, which is directly proportional to the protein content in a sample [32]. For estimating the relative abundance in a physiological state (fed or unfed) of proteins identified in the soluble and insoluble fractions the corresponding emPAI values were added.

In the Results section, redundant identifications were eliminated from the lists of identified proteins, in each case choosing the protein hit with the highest score. Keratins and other possible contaminants such as porcine trypsin were also excluded from the lists of proteins identified. In these results we added the additional identifications obtained in the EST_Acari database searches to the list of non-redundant proteins identified in the NCBInr_Metazoa database.

### Functional annotation and classification

Protein classification was performed according to the Gene Ontology (GO) hierarchy, using the Universal Protein Resource (UniProt) retrieval system (http://www.uniprot.org/) and the PANTHER (Protein ANalysis THrough Evolutionary Relationships) Classification System (http://www.pantherdb.org/) [33]. The “ID mapping” module for the UniProt system was used to transform the GI number to UniProt code, standardize protein symbols, and associate them with corresponding gene names, gene ontology categories and IDs, molecular function, subcellular location and biological process.

## Results

### Midgut protein extracts

Midguts from fed and unfed female ticks were homogenized and fractionated by centrifugation at 100,000 × g, obtaining two types of fractions: the fraction enriched in soluble proteins (supernatants S-0 and S-1) and the fraction enriched in insoluble membrane-associated proteins (pellets P-0 and P-1).

The proteins in each fraction were resolved by SDS-PAGE (Fig. 1). All fractions showed complex band patterns that covered a broad range of molecular sizes and revealed evident differences in protein composition between unfed and fed ticks (i.e., band patterns in the range of 52 kDa to 100 kDa). In the S-1 soluble fraction, obtained from fed specimens, the two more intense bands at 70 kDa and 15 kDa corresponded to the host serum albumin and haemoglobin, respectively. Fig. 1 also shows some interesting proteins among those identified in the different gel fragments in which each fraction was divided (see below).

The reproducibility of the sample preparations and fractionations was checked in three different batches of midguts that always showed band patterns identical to their homologous one in Fig. 1 (not shown).

### Proteins identified

In order to simplify the comparative study, we processed and analysed the Mascot results obtained from database searching with the combined spectra of the 10 gel slices from each fraction (S-0, P-0, S-1, P-1) (Fig. 1).

The results reported here refer to the identifications, on the basis of at least two significant peptides, performed with Mascot in the NCBInr_Metazoa and EST_Acari databases after removing redundancies and contaminants (Table 1). Since blood meal digestion in ticks is intracellular, all the fractions of tick midguts contained a mixture of tick proteins and host blood proteins. Thus, protein origin was assigned to the tick when the protein hit was from a tick, an arthropod or a non-mammalian vertebrate and to the host when the protein hit was from rabbit or any other mammalian species. Additional file 1: Table S1 and Additional file 2: Table S2 list the proteins of tick origin identified in unfed and fed *O. erraticus* females, respectively and Additional file 3: Table S3 lists the proteins of host origin. Additional file 4: Figure S1 represents the percentage of amino acid sequence coverage for all proteins identified in each fraction (supernatants and pellets).

**Table 1:** Number of unique proteins identified in the midgut fractions from *Ornithodoros erraticus* fasted females (unfed group) and from engorged females after 48 h post-feeding (fed group)

	Unfed ticks		Fed Ticks	
	Soluble fraction (S-0)	Insoluble fraction (P-0)	Soluble fraction (S-1)	Insoluble fraction (P-1)
Non-redundant proteins (n°): NCBI_Metazoa	124	223	242	319
NCBI_EST_Acari	138	284	138	261
Total non-redundant proteins	185	346	292	429
Tick proteins (n°)	158	330	141	291
N° of peptides	585	1287	535	1120
emPAI	72.7	229.5	62.39	203.41
Host proteins (n°)	27	16	151	138
N° of peptides	232	131	986	938
emPAI	685.4	376.0	3723.2	626.3

Redundant identifications and contaminants have been excluded. Soluble and insoluble fractions are the supernatants and pellets, respectively, after a centrifugation at 100.000 g of midgut homogenates

**Fig. 2:**
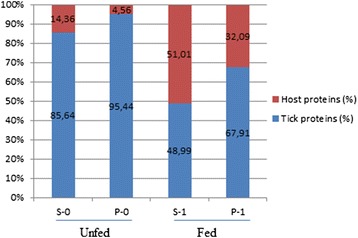
Ratio of unique proteins of either tick or host origin identified in each fraction obtained from midgut homogenates. S-1 and P-1, supernatant and pellet from midgut homogenates of fed ticks. S-0 and P-0, supernatant and pellet from midgut homogenates of unfed ticks

Regarding the proteins of host origin, Table 1 and Fig. 2 show their number and ratio in each fraction. As expected, host proteins were more numerous in the samples from fed ticks (51 and 32 %, in S-1 and P-1, respectively) than in the homologous samples from unfed ticks (14.4 and 4.6 %, in S-0 and P-0, respectively). These host proteins were abundantly represented in all the fractions, as indicated by the sums of their emPAI values. This was particularly evident in the case of the S-1 fraction, where rabbit haemoglobin and albumin accounted for most of the total emPAI value (3723.2) of the host proteins identified in this fraction (Fig. 1, bands of 15 and 70 kDa, respectively). In the present work these host proteins were omitted from further characterization and analysis.

Regarding the tick proteins, the number of non-redundant proteins identified in the different fractions ranged between 141 and 330, showing the insoluble fractions P-0 and P-1 the highest values (Table 1, Additional file 1: Table S1 and Additional file 2: Table S2).

**Fig. 3:**
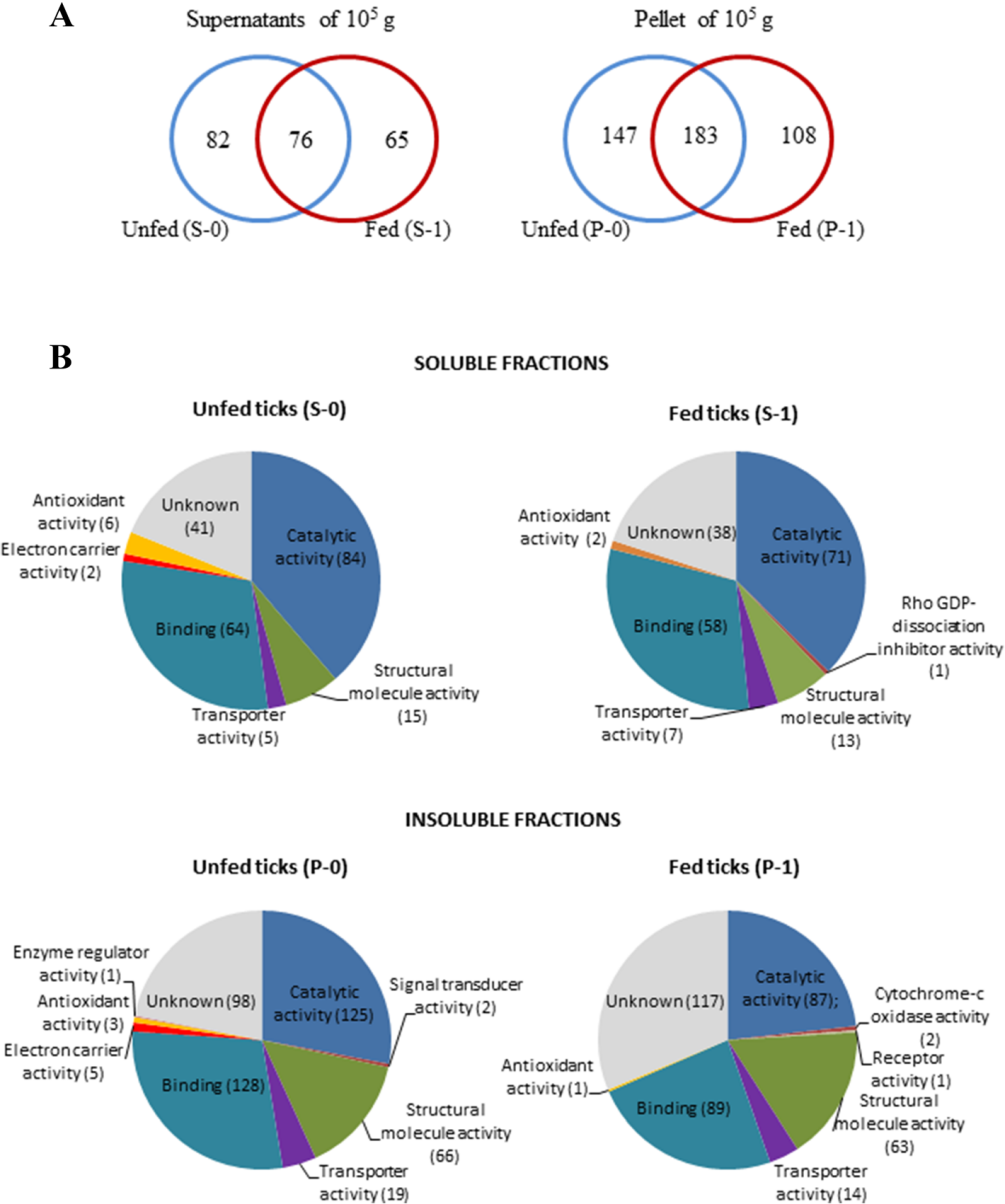
Proteins identified in the fractions. **a** Number of proteins identified in each of the fractions obtained from the midgut of fed and unfed ticks. S-0, S-1, P-0 and P-1, soluble (S) and insoluble fractions (P) from midgut of unfed and fed ticks. **b** Classification according to their molecular function of the proteins identified in each of the fractions

Proteomic analysis of the soluble fractions (S-0 and S-1) from unfed and fed ticks allowed the identification of a total of 223 non-redundant tick proteins: 82 only in the gut from unfed ticks; 65 only in fed ticks, and 76 in both groups (Fig. 3a). In the insoluble fractions (P-0 and P-1), 438 non-redundant proteins were identified: 147 only in the gut from unfed ticks; 108 only in fed ticks, and 183 in both groups of ticks (Fig. 3a).

The tick proteins identified in each fraction were classified according to their molecular function using the UniProt tools. As can be seen in Fig 3b, the functional classification was very similar for all four samples, whose proteins were distributed in the following categories: catalytic, binding, structural, transporter, antioxidant, electron carrier, GDP-dissociation inhibitor, cytochrome oxidase, and receptor activity. In all fractions (S-0, S-1, P-0, P-1), the most numerous proteins were those involved in catalytic (53.2 and 50.4 % in S-0 and S-1; 37.9 and 29.9 % in P-0 and P-1) and binding activities (40.5 and 41.1 % in S-0 and S-1; 38.8 and 30.6 % in P-0 and P-1). Structural proteins were also represented in all fractions, but they were twice more numerous in the insoluble than in the soluble fractions (20.0 and 21.6 % in P-0 and P-1 versus 9.5 and 9.2 % in S-0 and S-1). The remaining categories showed remarkably lower ratios in all fractions, except those classified as having an unknown molecular function, which ranged between 25.9 and 42.0 % (Fig. 3b). The available GO data on Molecular Function, Biological Process and Cellular Component for each protein are included in Additional file 1: Table S1 and Additional file 2: Table S2.

### Comparative analysis of the proteins identified in the midgut of unfed and fed *O. erraticus* females

**Fig. 4:**
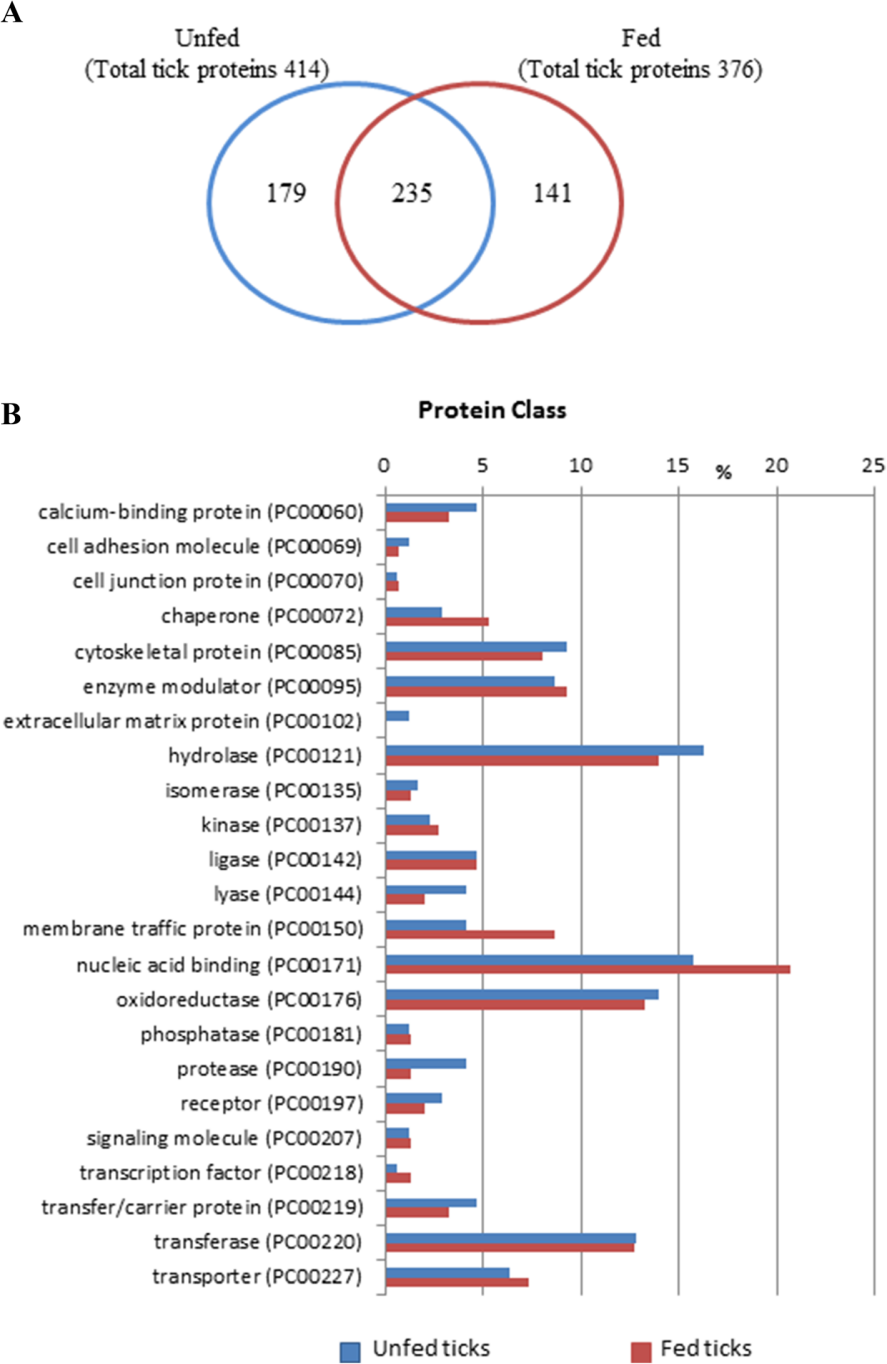
Proteins identified in the midgut of unfed ticks and engorged ticks at 48 h post-feeding. **a** Number of proteins identified in each experimental group, fed and unfed ticks. **b** The proteins identified in the midgut of unfed and fed ticks were classified into protein classes using the Panther Classification System. Bars represent the percentage of proteins in each protein class relative to the total number of proteins in the group

In order to perform a comparative analysis of the proteins identified in the midgut from the fed and unfed ticks, the two fractions from the same experimental group were grouped and analysed together. Overall, 555 non-redundant midgut proteins were identified: 414 in unfed ticks, 376 in fed ticks, and 235 in both groups, unfed and fed (Fig. 4a).

The proteins identified in each physiological state, unfed and fed, were classified by “Protein Class” using the Panther Classification System, which allowed the categorization of 172 genes/proteins from the unfed ticks and 150 genes/proteins from the fed ticks (Fig. 4b). For both proteomes, unfed and fed, Panther generated very similar distributions, in which the proteins were grouped within the same 23 protein classes. The most numerous protein classes were nucleic acid binding (15.7 % in unfed and 20.7 % in fed) and hydrolases (16.3 % in unfed and 14.0 % in fed), followed by oxidoreductases (14.0 % in unfed and 13.3 % in fed), transferases (12.8 % in unfed and 12.7 % in 12.7), enzyme modulators (8.7 % in unfed and 9.3 % in fed), cytoskeletal proteins (9.3 % in unfed and 8.0 % in fed), transporter (6.4 % in unfed and 7.3 % in fed) and membrane traffic proteins (4.1 % in unfed and 8.7 % in fed). There were few differences in the protein class ratios between the fed and unfed ticks, being membrane traffic and nucleic acid-binding proteins more numerous in the midgut of the fed ticks.

**Fig. 5:**
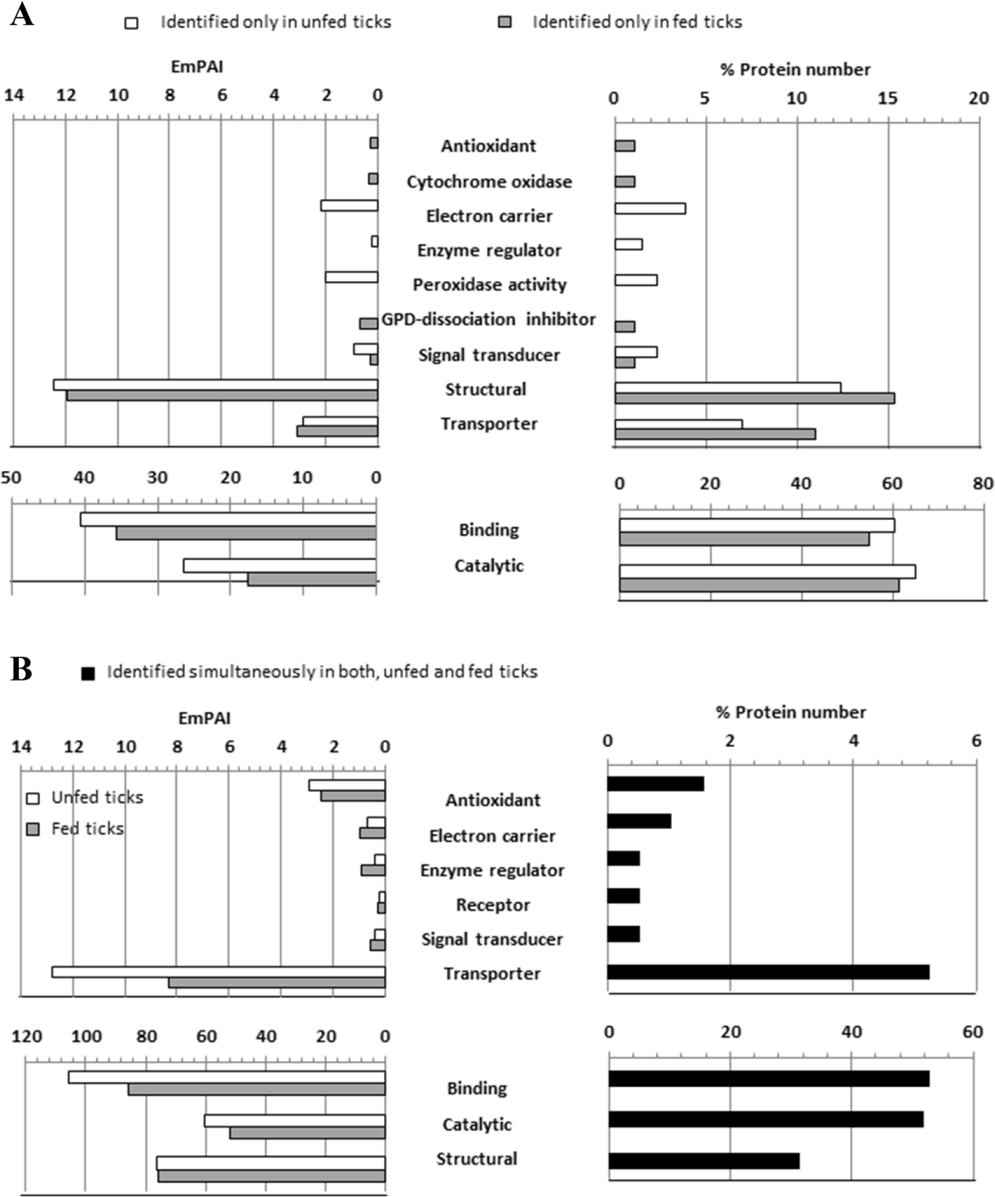
Classification by molecular function of the proteins identified either only in unfed ticks or only in fed ticks (**a**) and simultaneously in both groups of ticks (**b**). % Protein number is the ratio between the numbers of proteins identified in each category with respect to the total number of proteins classified. The emPAI value for each category was calculated as the sum of the emPAI of all the proteins in that category

Following this, the proteins identified only in unfed, only in fed and in both groups of ticks were analysed separately and categorized according to their “Molecular Function” using the tools available in the UniProt website (Fig. 5, Additional file 5: Table S4). This analysis revealed that the proteins involved in catalytic and binding activities were the most numerous and abundant proteins identified in the “only unfed” and “only fed” groups (Fig. 5a). The number of these catalytic and binding proteins was similar in both groups but the catalytic proteins were more abundant in the “only unfed” group (26.4 emPAI in unfed ticks versus 17.5 emPAI in fed ticks). Some additional differences were observed between both groups in the percentages of proteins with transporter (11.0 % only in fed versus 7.0 % only in unfed) and structural activity (15.4 % only in fed versus 12.4 % only in unfed), which were both more numerous in fed than in unfed ticks.

Regarding proteins identified simultaneously in unfed and fed ticks, the most numerous were those involved in binding, catalytic and structural functions. The main differences observed in this analysis were the higher abundance in unfed ticks of proteins with binding (105.8 emPAI in unfed versus 85.7 emPAI in fed) and transporter activity (12.8 emPAI in unfed versus 8.34 emPAI in fed) (Fig. 5b).

### Proteins involved in blood digestion and stress responses

Once having the global analysis of proteomes, we considered of interest to make a more in-depth comparison of the four functional groups of proteins most likely involved in the process of blood digestion and in other processes related to blood feeding. Consequently, we selected the following functional groups: (i) proteins with peptidase activity, (ii) proteins involved in iron metabolism and transport, (iii) proteins involved in responses to oxidative stress and detoxification associated with blood feeding and, (iv) proteins involved in endocytosis, membrane traffic and protein transport (Table 2 and Fig. 1).

**Table 2:** Proteins identified in the midgut from *Ornitodoros erraticus* females before feeding (unfed group) and after 48 h post-feeding (fed group) involved in the following biological activities and process: Peptidase activity, iron metabolism and transport, oxidoreductase, protein folding and response to stress and endocytosis, membrane traffic and protein transport

Experimental group	Entry	Gene names	Protein names	Code in Fig. 1	Function (Gene Ontology)	Num. of significant sequences		emPAI	
						Unfed	Fed	Unfed	Fed
Peptidase activity									
Unfed, Fed	Q2WFX6	AP	Aspartic protease	Pa1	Aspartic-type endopeptidase	2	2	0.32	0.32
Unfed, Fed	E7E820	-	Cathepsin D2	Pa2	Aspartic-type endopeptidase	2	3	0.34	0.26
Unfed, Fed	B7P6S9	IscW_ISCW000202	Tick legumain	Pa3	Cysteine-type endopeptidase	2	2	0.17	0.17
Unfed, Fed	F0J8F6	-	Metallopeptidase (Fragment)	Pa4	-	2	3	0.22	0.41
Unfed, Fed	B7PA58	IscW_ISCW003100	Putative uncharacterized protein	Pa5	Metalloexopeptidase	2	2	0.14	0.14
Unfed, Fed	Q6U8A8	-	Serine protease-like protein	Pa6	Serine-type endopeptidase	4	4	0.96	0.75
Unfed, Fed	Q09JL3	-	Signal peptidase complexI	Pa7	Serine-type peptidase	5	2	1.28	0.39
Unfed	E2BXE8	EAI_04817	AFG3-like protein 2	Pa8	Metalloendopeptidase	4	-	0.51	-
Unfed	B7Q203	IscW_ISCW009180	ATPase	Pa9	Metalloendopeptidase	3	-	0.26	-
Unfed	B7P573	IscW_ISCW001592	Processing peptidase beta subunit,	Pa10	Metalloendopeptidase	2	-	0.13	-
Unfed	B4NAG0	Dwil\GK11711	GK11711	Pa11	Peptidase activity	2	-	0.36	-
Unfed	B7PXW8	IscW_ISCW020703	Signal peptidase complex, subunit SPC25	Pa12	Peptidase activity	2	-	0.31	-
Fed	B7Q579	IscW_ISCW021356	Acylamino-acid-releasing enzyme	Pa13	Serine-type peptidase	-	2	-	0.08
Fed	M7CBK1	UY3_00674	Protein DDI1 like protein 2	Pa14	Aspartic-type endopeptidase	-	2	-	0.06
Fed	A0A087UYK2	X975_16479	Signal peptidase complex catalytic subunit SEC11C	Pa15	Serine-type peptidase	-	2	-	0.21
Iron metabolism and transport									
Unfed, Fed	B9UNL8	-	Aconitate/iron-regulatory protein	Ir1	4 iron, 4 sulfur cluster binding	2	3	0.3	0.1
Unfed, Fed	B7P592	IscW_ISCW015613	Amidophosphoribosyltransferase (ATase)	Ir2	Iron-sulfur cluster binding	2	3	0.24	0.19
Unfed, Fed	O99806	-	Cytochrome c oxidase subunit 1	Ir3	Iron ion binding	2	2	0.12	0.38
Unfed, Fed	A6N9Q6	-	Ferritin	Ir4	Iron ion transport	2	2	0.36	0.36
Unfed	Q16LR5	AAEL012552	AAEL012552-PA	Ir5	Iron-sulfur cluster binding	3	-	0.13	-
Unfed	J3JUT5	YQE_06758	Cytochrome b-c1 complex subunit Rieske	Ir6	2 iron, 2 sulfur cluster binding	2	-	0.24	-
Unfed	F4WQE0	G5I_08058	NADH dehydrogenase [ubiquinone] iron-sulfur protein	Ir7	4 iron, 4 sulfur cluster binding	2	-	0.2	-
Unfed	B7PNX4	IscW_ISCW005985	NADH:ubiquinone oxidoreductase, NDUFV1/51 kDa subunit,	Ir8	4 iron, 4 sulfur cluster binding	2	-	0.13	-
Unfed	B7PBH7	IscW_ISCW003299	NADH-ubiquinone reductase	Ir9	Iron-sulfur cluster binding	2	-	0.22	-
Unfed	A7RKR4	v1g179073	Predicted protein	Ir10	4 iron, 4 sulfur cluster binding	3	-	0.37	-
Unfed	Q86GF8	-	Putative uncharacterized protein	Ir11	4 iron, 4 sulfur cluster binding	2	-	0.08	-
Unfed	G6D2B9	KGM_16016	Uncharacterized protein	Ir12	4 iron, 4 sulfur cluster binding	3	-	0.38	-
Unfed	T1FN77	HELRODRAFT_185731	Uncharacterized protein	Ir13	Iron-sulfur cluster binding	3	-	0.13	-
Oxidoreductase, protein folding and response to stress									
Unfed, Fed	A1KXI6	-	Blo t aldehyde dehydrogenase allergen	Ox1	Oxidoreductase	2	3	0.44	0.49
Unfed, Fed	B7P4E1	IscW_ISCW000393	Glutamate dehydrogenase	Ox2	Oxidoreductase	3	3	0.17	0.28
Unfed, Fed	B7QKE4	IscW_ISCW023707	Glycerol-3-phosphate dehydrogenase	Ox3	Oxidoreductase	2	2	0.08	0.18
Unfed, Fed	Q4PLZ0	-	Mitochondrial malate dehydrogenase	Ox4	Oxidoreductase	2	6	0.35	1.83
Unfed, Fed	Q09JE3	-	Superoxide dismutase [Cu-Zn]	Ox5	Oxidoreductase	3	3	1.87	1.36
Unfed, Fed	A6N9S1	-	Thioredoxin peroxidase	Ox6	Oxidoreductase	6	3	1.92	0.54
Unfed, Fed	A6NA14	-	Truncated peroxiredoxin	Ox7	Oxidoreductase	3	3	0.55	0.55
Unfed, Fed	F0J8S6	-	FKBP-type peptidyl-prolyl cis-trans isomerase	Ox8	Protein folding	3	2	0.35	0.26
Unfed, Fed	B2ZWT4	CyPA	Peptidyl-prolyl cis-trans isomerase	Ox9	Protein folding	3	3	0.84	0.84
Unfed, Fed	A0A087UTZ9	X975_09861	T-complex protein 1 subunit alpha	Ox10	Protein folding	2	3	0.11	0.4
Unfed, Fed	E9GYM5	DAPPUDRAFT_306806	T-complex protein 1 subunit gamma	Ox11	Protein folding	3	3	0.17	0.33
Unfed, Fed	F0J8P3	-	HSP70 family member	Ox12	Protein folding/response to stress	12	11	4.0	3.5
Unfed, Fed	B7QI01	IscW_ISCW014265	Hsp90 protein	Ox13	Protein folding/response to stress	16	21	1.17	3.03
Unfed, Fed	B7QC85	IscW_ISCW022766	Tumor rejection antigen (Gp96),	Ox14	Protein folding/response to stress	5	9	0.95	1.46
Unfed, Fed	Q2YFF0	-	Glutathione transferase mu class	Ox15	Response to stress and detoxification	2	2	0.27	0.25
Unfed	B7P0P1	IscW_ISCW016225	DNA topoisomerase 2	Ox16	Oxidoreductase	2	-	0.38	-
Unfed	B7QBE5	IscW_ISCW013475	Lipophorin receptor	Ox17	Oxidoreductase	2	-	0.27	-
Unfed	Q2XW18	PHGPX	Glutathione peroxidase	Ox18	Response to oxidative stress	4	-	1.12	-
Fed	A0A087U1A2	X975_14796	Copper chaperone for superoxide dismutase	Ox19	Oxidation-reduction process	-	2	-	0.24
Fed	Q6DJQ3	aldh9a1 TEgg018l09.1-001	Aldehyde dehydrogenase 9 family, member A1	Ox20	Oxidoreductase	-	2	-	0.12
Fed	B7Q8W6	IscW_ISCW010532	Alkyl hydroperoxide reductase, thiol specific antioxidant	Ox21	Oxidoreductase	-	2	-	0.27
Fed	U6PA25	HCOI_01121300	Endonuclease exonuclease phosphatase	Ox22	Oxidoreductase	-	2	-	0.07
Fed	I1WDI0	-	Putative 17 beta-hydroxysteroid dehydrogenase	Ox23	Oxidoreductase	-	2	-	0.34
Fed	E9HGS0	DAPPUDRAFT_228809	Uncharacterized protein	Ox24	Oxidoreductase	-	2	-	0.22
Fed	T1FVK5	HELRODRAFT_194011	Uncharacterized protein	Ox25	Oxidoreductase	-	2	-	0.12
Fed	B7QJ21	IscW_ISCW023397	Chaperonin complex component, TCP-1 eta subunit,	Ox26	Protein folding	-	2	-	0.21
Fed	Q0ZBX3	AG-0383 F-Gp	Putative accessory gland protein	Ox27	Protein folding	-	2	-	0.24
Fed	A0A067RDC2	L798_09307	T-complex protein 1 subunit delta	Ox28	Protein folding	-	2	-	0.37
Fed	F0J987	-	Endoplasmic reticulum glucose-regulated protein	Ox29	Protein folding/response to stress	-	4	-	0.67
Endocytosis, membrane traffic and protein transport									
Unfed, Fed	B7QHS0	IscW_ISCW015012	Flotillin	Et1	_	4	2	0.33	0.14
Unfed, Fed	B7P427	IscW_ISCW001550	Transmembrane protein Tmp21	Et2	Protein transport	2	2	0.48	0.30
Unfed, Fed	B7P6P0	IscW_ISCW001001	Glycoprotein 25 l	Et3	Protein transport	3	3	0.63	0.44
Unfed, Fed	B7PUK8	IscW_ISCW019441	Clathrin heavy chain	Et4	Vesicle-mediated transport	12	10	0.25	0.21
Unfed, Fed	B7PQE0	IscW_ISCW006283	Coatomer, alpha chain	Et5	Vesicle-mediated transport	2	2	0.06	0.06
Unfed, Fed	B7PDY5	IscW_ISCW004922	Vesicle coat complex COPII, GTPase subunit SAR1	Et6	Vesicle-mediated transport	3	3	0.53	0.60
Unfed	B7Q4N0	IscW_ISCW011849	Cargo transport protein EMP24	Et7	Protein transport	2	-	0.22	-
Unfed	B7PGX4	IscW_ISCW003940	Synaptic vesicle-associated integral membrane protein	Et8	Vesicle-mediated transport	4	-	0.65	-
Fed	B7QNW0	IscW_ISCW015531	Protein required for fusion of vesicles in vesicular transport, alpha-SNAP	Et9	Protein transport	-	3	-	0.35
Fed	B7PYR6	IscW_ISCW020077	Protein transport protein SEC61 alpha subunit	Et10	Protein transport	-	4	-	0.32
Fed	B7P806	IscW_ISCW016976	Vesicle docking protein P115	Et11	Protein transport	-	2	-	0.08
Fed	B7Q6V9	IscW_ISCW011108	AP-2 complex subunit beta-1	Et12	Vesicle-mediated transport	-	5	-	0.17
Fed	B7QAA7	IscW_ISCW012850	Coatomer beta subunit	Et13	Vesicle-mediated transport	-	3	-	0.12
Fed	B7QCH5	IscW_ISCW022475	Coatomer gamma subunit	Et14	Vesicle-mediated transport	-	3	-	0.14
Fed	B7Q5G4	IscW_ISCW021581	Endocytosis/signaling protein EHD1	Et15	Vesicle-mediated transport	-	2	-	0.18

We identified 15 proteins with peptidase activity, 13 in unfed ticks and 9 in fed ticks, of which 7 were identified in both groups. These proteases belonged to four different groups (aspartic-type endopeptidase, cysteine-type endopetidase, metallopeptidase and serine-type endopeptidase), except two of them, in which the type of enzyme activity has still not been characterized (B4NAG0, B7PXW8). No predominance of one or another type of molecule was observed as a function of the physiological conditions of the tissue analyzed and we only detected a lower abundance of the signal peptidase complex I protein in the fed ticks (1.28 emPAI in unfed versus 0.39 emPAI in fed).

Regarding the 13 proteins related to iron metabolism and transport, all of these were present in the midgut of unfed ticks and only 4 were also identified in fed ticks. These latter were aconitase, ATPase, cytochrome c oxidase and ferritin, without differences in the emPAI values between fed and unfed ticks (Table 2). The other proteins identified only in unfed specimens were the AAEL012552-PA (Q16LR5) protein and an uncharacterized protein (T1FN77), which shared 72–93 % identity with an NADH-ubiquinone oxidoreductase; cytochrome b-c1 complex; NADH dehydrogenase iron-sulphur protein; two NADH-ubiquinone reductases; a predicted protein (A7RKR4) and two uncharacterized proteins (Q86GF8, G6D2B9), which shared 82 % and 84 % identity with an aconitase hydratase.

We also have identified, mainly in fed ticks, 29 proteins involved in responses to oxidative stress and detoxification associated with blood feeding. As can be seen in Table 2, 15 were identified in the midgut of fed and unfed ticks, three only in unfed ticks and 11 only in fed ticks. According to the classification in the GO database, 11 of these proteins -chaperones of the T-complex protein 1, HSP70, HSP90, Gp96, the accessory gland protein, two peptidyl-propyl isomerases, and endoplasmic reticulum glucose regulated protein- could be involved in protein folding processes associated with stress responses. The other 18, among them glutathione peroxidase, thioredoxin peroxidase, superoxide dismutase, aldehyde dehydrogenase and others, could act as antioxidants in processes of detoxification and responses to oxidative stress.

Regarding intracellular blood digestion, it has been proposed that haemoglobin recognition and trafficking within tick digestive cells utilizes molecular mechanisms analogous to the clathrin-dependent receptor-mediated endocytosis of mammalian cells [16]. Table 2 shows 15 proteins involved in endocytosis processes (clathrin, flotillin, AP-2 complex, endocytosis/signalling protein EHD1) intracellular protein transport (SEC61, cargo transport protein EMP24, glycoprotein 25I, transmembrane protein TMP21) and vesicle-mediated transport (cotoamer complex, alpha SNAP, vesicle-docking protein P115, synaptic vesicle-associated protein, vesicle coat complex COPII). Most of them (13 proteins) were found in the midgut of fed *O. erraticus* females, and six of them in both fed and unfed ticks. The latter showed similar emPAI values in both physiological states, suggesting that their expression level does not change after blood feeding.

## Discussion

The midgut of ticks is a particularly promising target for the development of new control strategies. The luminal surface of the midgut can be accessed to by the host immune effectors and blood components ingested during blood feeding. Additionally, since blood meal digestion in ticks is intracellular, blood components may also enter midgut cells [22, 34]. Therefore, vaccine-induced antibodies and drugs present in host blood could reach their targets in the tick midgut after blood feeding. Proof of this is that the only two commercialized anti-tick vaccines available are based on an intestinal antigen [35, 36].

It has also been demonstrated that the ingestion of drugs present in blood may have a deleterious effect on ticks. An example of this is the effect of the recently commercialized drug Fluralaner (Bravecto™), although it does not target intestinal proteins, its oral administration has proved to be effective against several tick species, causing their death a few hours after feeding [37–39].

In light of the above, it is clear that the knowledge of the gut proteome and changes in protein expression upon feeding and digestion provides key information for identifying and selecting new targets for the development of alternative control strategies. Accordingly, the aim of the present work was to construct the intestinal proteome at two moments in the trophogonic cycle of the tick, namely, unfed and at 48 h post-feeding. These sampling time-points were selected considering the phases of digestion in argasids, in order to analyze midgut tissues under basal conditions and during the process of digestion. In argasids, digestion begins as soon as they detach themselves from the host and, as has been described in *O. moubata*, it comprises three phases whose duration depends on environmental and physiological factors [17, 18]. During the first hours post-feeding, blood begins to become concentrated, excess water and sodium ions being expelled through the coxal glands; then, erythrocyte haemolysis begins, and the digestion of haemoglobin is insignificant. The second stage (2–5 days) is the phase of intensive digestion, with uptake of the blood meal components into enterocytes, their digestion, and the elimination of residues. The third phase may be a long period of very slow digestion, enabling the tick to fast for long periods of time [17, 18]. Moreover, in *O. erraticus* we had previously observed that the expression of particular intestinal antigens, even though they are expressed constitutively along the trogophonic cycle, increases significantly after a blood meal, with a maximum at 24–72 h post-detachment [11].

In the present work the protocol used for sample collection and pre-processing for mass spectrometry analysis involved sample fractionation by centrifugation followed by separation of the proteins in each fraction by SDS-PAGE. Bearing in mind that in ticks collected after feeding on a host the major constraint for the successful identification of tick proteins is a large amount of host protein [40], we sliced the gel into pieces and analysed each gel piece individually. In this way, the very abundant host proteins, such as haemoglobin and albumin, were concentrated in a few gel pieces, thus preventing these proteins from masking the detection of most tick proteins in the remaining gel pieces. Proof of this is the fact that the numbers of non-redundant tick proteins identified in the midgut of the fed and unfed females were similar: 376 and 414 respectively.

Overall, the proteomic analyses of the midgut of unfed and fed ticks identified 555 non-redundant tick proteins. The analysis of Molecular Function gene ontology showed a significant proportion of proteins with an unknown function in all fractions from both groups (Fig. 3b). This was to a certain extent expected because *O. erraticus* is a non-model species and its genome is basically unknown. Apart from the proteins with unknown function, those most represented in all fractions were proteins with catalytic and binding activity (Fig. 3b), consistent with their role in the intracellular processing of the blood meal [21]. A notably high proportion of structural proteins was identified in both unfed and fed ticks, mainly in the P-0 and P-1 insoluble fractions, most of these proteins being structural constituents of ribosomes, such as ribosomal RNA 40S, 60S, and other ribosomal genes involved in protein synthesis [21, 41]. Moreover, it was also suggested that ribosomes would serve as hub for translational folding, chaperone interaction, degradation, and stress response [42].

Comparison of the midgut proteomes of unfed and fed ticks did not reveal any great differences in either the number or the type of proteins identified, as may be inferred from the classifications based on Molecular Function and Protein class (Figs. 4 and 5). These results were not unexpected since in similar studies performed in ixodid ticks it was observed that the composition of the midgut proteome is highly stable during the early phase of feeding [25, 41]. By contrast, at transcriptome level important changes in gene expression are seen in response to tick feeding; in particular, most of the proteins involved in blood digestion are upregulated. According to other authors, this suggests that post-transcriptional and post-translational regulation mechanisms can likely make proteome and transcriptome dynamics to have different kinetics, avoiding a direct correlation between mRNA and protein level [21, 24, 25, 41].

However, after a more detailed analysis of certain groups of proteins identified in *O. erraticus* putatively involved directly in blood meal digestion -including protein digestion (peptidase activity), iron metabolism, enzymes involved in oxidative stress and detoxification and membrane traffic and transport- we detected some differences between the fed and unfed ticks. It should be noted that some of the differences observed in protein composition could in fact represent quantitative differences in the expression level of the proteins, since the least abundant proteins would be below the threshold of detection by MS.

The pathway of haemoglobin degradation in ixodids proceeds via the generation of large initial fragments (8–11 kDa) to smaller haemoglobin-derived peptides (2–7 kDa), which are finally hydrolysed to dipeptides and free amino acids [16]. The degradation pathway is initiated by endopeptidases of the aspartic and cysteine classes (cathepsin D supported by cathepsin L and legumain), after which a cathepsin B participates in the production of smaller fragments, and finally the pool of peptide fragments is degraded into dipeptides and amino acids through the action of cathepsin C, cathepsin B, a carboxipeptidase and a leucine aminopeptidase [16, 43]. In argasids, information about the machinery of blood digestion is very scant and limited to a previous description of protease activity in the midgut of *O. tolozani* [44] and to the more recent identification of two cystatins in *O. moubata* [29]. In *O. erraticus* we have identified 15 proteins with peptidase activity, one of which is a cathepsin D2 and a legumain. We also identified several proteins with metalloprotease activity, some of which could exert functions similar to that of leucine aminopeptidase, since they belong to the same enzyme class (metallopeptidase class) [16, 45]. All of these proteins could be responsible for the cleavage of the haemoglobin molecule in spite of other important function like midgut cellular integrity/remodelling and embryogenesis [43, 46].

During blood digestion, ticks are exposed to an enormous amount of free iron, which must be appropriately used and detoxified. Whereas iron is an essential component of several proteins involved in fundamental biochemical activities and an essential nutrient for reproduction and embryonic development, it is also potentially toxic owing to its ability to generate reactive oxygen species [47]. For this reason, iron homeostasis must be tightly regulated by an orchestrated set of proteins that govern iron uptake, utilization, transport and storage [48]. Here we have identified 13 proteins classified as iron-binding proteins in the gene ontology database. All of them were expressed in the intestine of unfed females and only four also in fed females. This suggests a putative decreased expression of this protein group during blood feeding, an effect also observed by Anderson et al. [21] in the intestinal transcriptome of *Dermacentor variabilis*. In hard ticks it has been demonstrated that iron-regulatory proteins and ferritin play important roles in iron metabolism. Iron-regulatory proteins mediate the translational control of ferritin in response to iron levels [47]. Ferritins are crucial antioxidant molecules that protect hard ticks from iron-mediated oxidative stress during blood feeding, and have shown promising results as vaccine antigens against tick infestation [47–51]. In the *O. erraticus* females we found an aconitase/iron-regulatory protein and a homologue of the *O. parkeri* ferritin, whose expression in the midgut did not change after a blood meal. A similar result has been reported for *Haemaphysalis longicornis* ferritins [52].

We also identified several chaperones and antioxidant proteins with oxidoreductase activity, probably involved in stress responses and detoxification reactions associated with blood feeding. In this group, we identified 29 proteins, most of which were found in fed ticks; this could indicate, as in the case of the mialome of *Dermacentor marginatus* [21], that the expression of this protein group increases during blood feeding. We have identified several antioxidant enzymes such as GSTs, thioredoxins, glutathione peroxidase and superoxide dismutase (SOD) which have already been identified in the midgut of several ixodid species, where they are known to play an important role in cellular stress responses such as those occurring as a result of blood feeding as well as in innate immunity [21, 23, 53, 54]. Interestingly SOD, which functions as an antioxidant by scavenging free radicals, appears to bind haeme. This suggests that in addition to its antioxidant properties it could function in haeme trafficking, which would be important in the intracellular tick blood meal digestion process [21, 55].

Regarding the intracellular blood digestion, it is proposed that haemoglobin recognition and trafficking in digestive cells utilize molecular mechanisms analogous to the clathrin-dependent receptor-mediated endocytosis of mammalian cells [16]. In *O. erraticus* we found 15 proteins (most of them in fed midguts) that may play some role in directing macromolecules into midgut cells and in intracellular protein transport. Clathrin and coatomer proteins are required to coat vesicles that are important for cargo selection and the direction of transfer [56]. The AP-2 complex belonging to the adaptin family, also identified in the midgut of *Rhipicephalus microplus*, mediates endocytosis by the plasma membrane and is part of the vesicle coat [23, 57]. TMP21 and related proteins, such as glycoprotein 251 and the cargo transport protein EMP24, are major membrane components of COPI- and COPII-coated vesicles and are involved in the endoplasmic reticulum to Golgi transport [58, 59]. Flotillins have been implicated in numerous processes, including endocytosis, signal transduction and regulation of the cortical cytoskeleton. However, the molecular mechanisms that underlie flotillin function in these different cases are still poorly understood [60]. According to Kongsuwan et al. [23] the evidence suggests that the transport machineries in tick midguts are complex and tightly regulated and the major challenge now is to understand the roles of these proteins in tick gut function.

## Conclusions

In this study we report for the first time the collection and analysis of the midgut proteome of an argasid tick species. This analysis includes a comparison of proteomic changes in response to tick feeding and blood digestion, providing hitherto unknown molecular information about the machinery of argasids for blood digestion. Analysis of the corresponding transcriptomes will likely increase and complement this information, allowing a more in-depth understanding of the biochemistry and physiology of blood digestion. This information could be a starting point for the identification and selection of new targets for the development of alternative control strategies.

## Additional files

Additional file 1: Table S1. Non-redundant tick proteins identified in the midgut of unfed ticks. (XLSX 78 kb) Additional file 2: Table S2. Non-redundant tick proteins identified in the midgut of engorged ticks at 48 h post-feeding (fed group). (XLSX 115 kb) Additional file 3: Table S3. Non-redundant host proteins identified in Soluble fraction, S-1, from midgut of engorged ticks at 48 h post-feeding (fed group). (XLSX 45 kb) Additional file 4: Figure S1. Pie charts showing the amino acid coverage for all the identified protein with two unique significant peptides in the different midgut fractions. The identified proteins in each fraction after mining either the BCBInr_metozoa or the EST_acari databases are included. For each chart, the proteins are grouped according to the sequence coverage into the following ranges: < 10 %, 10–25 %, 25–40 %, 40–60 % and > 60 %. For each sequence coverage range, the percentage of grouped proteins is given. (TIFF 104 kb) Additional file 5: Table S4. Non-redundant proteins identified only in unfed ticks, classified by their molecular function using the UniProt tools. (XLSX 88 kb)
