# Supplementary figures and images for: Midgut proteome of an argasid tick, Ornithodoros erraticus: a comparison between unfed and engorged females
Source: Parasit Vectors. 2015 Oct 12;8:525. doi: 10.1186/s13071-015-1148-z (PMC4603979; doi:10.1186/s13071-015-1148-z)

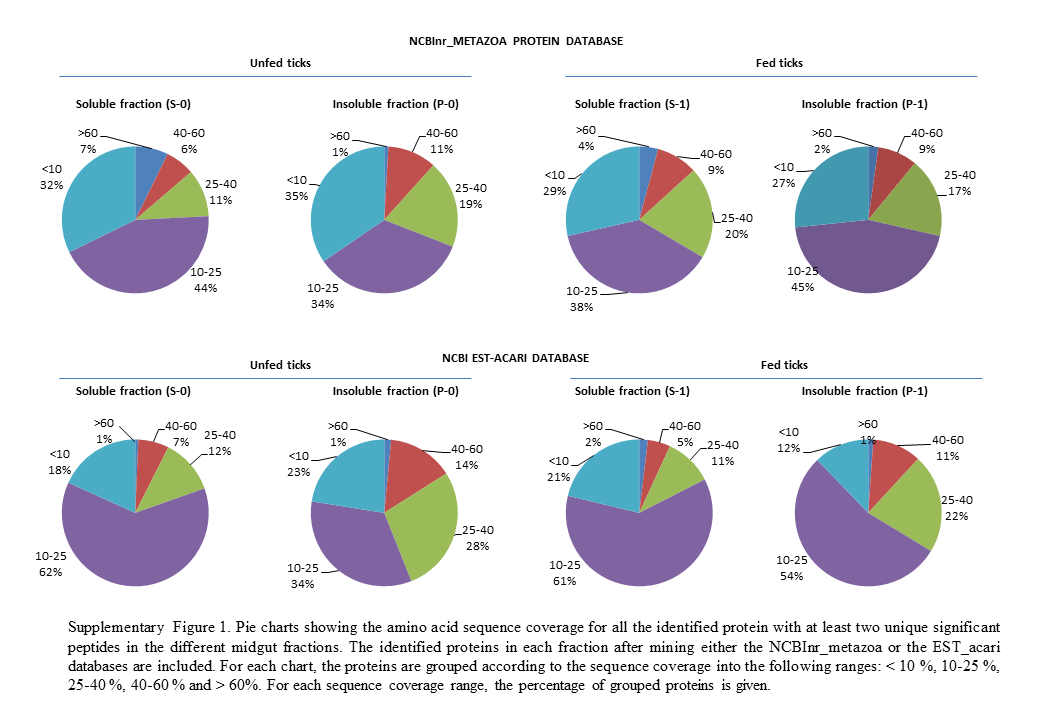

Supplement: Additional file 4: Figure S1. — Pie charts showing the amino acid coverage for all the identified protein with two unique significant peptides in the different midgut fractions. The identified proteins in each fraction after mining either the BCBInr_metozoa or the EST_acari databases are included. For each chart, the proteins are grouped according to the sequence coverage into the following ranges: < 10 %, 10–25 %, 25–40 %, 40–60 % and > 60 %. For each sequence coverage range, the percentage of grouped proteins is given. (TIFF 104 kb) [file 13071_2015_1148_MOESM4_ESM.tif]
